# Prolonged grief disorder in ICD-11 and DSM-5-TR: differences in prevalence and diagnostic criteria

**DOI:** 10.3389/fpsyt.2024.1266132

**Published:** 2024-02-08

**Authors:** Julia Treml, Katja Linde, Elmar Brähler, Anette Kersting

**Affiliations:** ^1^ Department of Psychosomatic Medicine and Psychotherapy, Medical Faculty, University of Leipzig, Leipzig, Germany; ^2^ Department of Psychosomatic Medicine and Psychotherapy, University Medical Center, Johannes Gutenberg-University Mainz, Mainz, Germany; ^3^ Department of Medical Psychology and Medical Sociology, University of Leipzig, Leipzig, Germany

**Keywords:** prolonged grief disorder, ICD-11, DSM-5, bereavement, prevalence

## Abstract

**Background:**

Prolonged grief disorder (PGD) was recently included as a disorder in the ICD-11 and DSM-5-TR. Although both classification systems use the same name, the criteria content, and diagnostic approach vary. This study aimed to estimate the respective prevalence of PGD_ICD-11_ and PGD_DSM-5-TR_ and examine the diagnostic agreement while varying the diagnostic algorithm of PGD_ICD-11_ (bereavement vs. symptom period; varying number of accessory symptoms).

**Methods:**

A representative sample of the German general population (N = 2,509) was investigated, of which n=1,071 reported the loss of a close person. PGD symptoms were assessed with the Traumatic Grief Inventory - Self Report Plus (TGI-SR+).

**Results:**

The point prevalence of PGD among the bereaved varied between 4.7%-6.8%, depending on the criteria and diagnostic algorithm. The prevalence of PGD_DSM-5-TR_ was significantly lower than the prevalence of PGD_ICD-11_. The diagnostic agreement between both criteria sets was substantial and increased after the number of accessory symptoms for PGD_ICD-11_ was increased from one to three. The most common symptoms were intrusive thoughts/images related to the deceased person, longing for the deceased person, and difficulty accepting the loss.

**Conclusion:**

The results demonstrate that the prevalence of PGD significantly varies depending on the application of the diagnostic algorithm and criteria. PGD affects a substantial proportion of the general population and should be addressed by healthcare providers. However, applying the minimum ICD-11 criteria could lead overestimating the prevalence. Therefore, further harmonizing the ICD-11 and DSM-5-TR criteria and diagnostic algorithm for PGD seems appropriate.

## Introduction

Losing a loved one is often accompanied by intense feelings of grief and longing for the deceased. Most bereaved people are able to adapt to the loss over time without professional support (1). However, when grief reactions persist and impede daily functioning, a disorder called *Prolonged Grief Disorder* (PGD) should be considered.

The conceptualization, diagnostic criteria, and assessment of PGD have been the topics of debate among researchers for many years (2). Some have called for the inclusion of PGD in the International Statistical Classification of Diseases and Related Health Problems (ICD) and the Diagnostic and Statistical Manual of Mental Disorders (DSM) while proposing different diagnostic criteria-sets [e.g., (3–5)]. The DSM-5 workgroup on Trauma/Stress-Related and Dissociative Disorders first decided to include PGD as *Persistent Complex Bereavement Disorder* (PCBD) in Section III, “Conditions for Further Study” within the DSM-5 to encourage research into the condition (2, 6, 7). Later, the ICD-11 workgroup on Stress-Associated Disorders found the evidence for PGD sufficient and included PGD in the ICD-11 as a new diagnostic entity (8). Based on this inclusion and the collected evidence, the DSM Steering Committee reviewed a proposal to modify the criteria for PCBD. The American Psychiatric Association (APA) then approved the inclusion of PGD in Section II of the DSM-5-TR, thus replacing the criteria for PCBD (9, 10).

Although both classification systems included PGD as a disorder and used the same name, the criteria content and diagnostic approach vary. While the DSM-5-TR provides an explicit diagnostic algorithm, the ICD-11 only uses a typological approach without strict requirements for the number of symptoms that must be present to meet the diagnostic threshold (11). **Table 1** provides an overview of the diagnostic criteria. A PGD_DSM-5-TR_ diagnosis requires *one of two* separation distress symptoms (persistent and pervasive longing for the deceased and/or persistent and pervasive preoccupation with the deceased), and at least *three out of eight* accessory symptoms to a functionally impairing degree, with a minimum of 12 months since the loss (12). A PGD_ICD-11_ diagnosis, however, only requires at least *one of two* separation distress symptoms, combined with *any* of ten accessory symptoms to a functionally impairing degree for an atypically long period of time (at least six months) after the loss (8).

**Table 1:** Diagnostic criteria for PGD.

ICD-11*	DSM-5-TR
A. Disturbance following the death of a partner, parent, child, or other person close to the bereaved	A. The death of a person close to the bereaved *at least 12 months* previously
B. Persistent and pervasive grief response characterized by longing for the deceased or persistent preoccupation with the deceased	B. Since the death, there has been a grief response characterized by *one or both* of the following, to a clinically significant degree, nearly every day or more often for *at least the last month*: 1. Intense yearning/longing for the deceased person 2. Preoccupation with thoughts or memories of the deceased person
C. Accompanied by intense emotional pain, e.g.: 1. Sadness 2. Guilt 3. Anger 4. Denial 5. Blame 6. Difficulty accepting the death 7. Feeling one has lost a part of one’s self 8. An inability to experience positive mood 9. Emotional numbness 10. Difficulty in engaging with social or other activities	C. As a result of the death, at least 3 of the following 8 symptoms have been experienced to a clinically significant degree since the death, including nearly every day or more often for at least the last month: 1. Identity disruption (e.g., feeling as though part of oneself has died 2. Marked sense of disbelief about the death 3. Avoidance of reminders that the person is dead 4. Intense emotional pain (e.g., anger, bitterness, sorrow) related to the death 5. Difficulty with reintegration into life after the death (e.g., problems engaging with friends, pursuing interests, planning for the future) 6. Emotional numbness (i.e., absence or marked reduction in the intensity of emotion, feeling stunned) as a result of the death 7. Feeling that life is meaningless as a result of the death 8. Intense loneliness (i.e., feeling alone or detached from others) as a result of the death
D. The grief response has persisted for an atypically long period of time following the loss (more than 6 months at a minimum) and clearly exceeds expected social, cultural or religious norms for the individual’s culture and context. Grief reactions that have persisted for longer periods that are within a normative period of grieving given the person’s cultural and religious context are viewed as normal bereavement responses and are not assigned a diagnosis.	D. The disturbance causes clinically significant distress or impairment in social, occupational, or other important areas of functioning.
E. The disturbance causes significant impairment in personal, family, social, educational, occupational or other important areas of functioning.	E. The duration and severity of the bereavement reaction clearly exceeds expected social, cultural, or religious norms for the individual’s culture and context
	F. The symptoms are not better explained by major depressive disorder, posttraumatic stress disorder, or another mental disorder, or attributable to the physiological effects of a substance (e.g., medication, alcohol) or another medical condition.

*ICD-11 criteria were ordered by the authors analogous to the DSM-5-TR criteria for better comparability.

Some researchers have argued that the ICD-11’s typological approach, with simple diagnostic descriptions and no strict requirement for the number of symptoms needed to meet a diagnostic threshold, has the advantage of increasing cross-cultural applicability and being helpful in clinical practice as it results in greater sensitivity in case identification (13). Others, however, criticize this approach for being too liberal since the application of the minimal criteria leads to much higher prevalence rates than the prior used criteria sets for PGD (such as PCBD) or the PGD_DSM-5-TR_ criteria (11). For instance, Boelen and colleagues compared the PGD_ICD-11_ criteria to the former PCBD_DSM-5_ criteria and found prevalence rates of 18% vs. 6.4%, respectively. Furthermore, Rosner and colleagues found prevalence rates of 4.2% for PGD_ICD-11_ compared to 3.3% for PGD_DSM-5-TR_ in a representative bereaved sample in Germany (14). A recent study extended these findings and demonstrated limited content overlap between both criteria sets and their predecessors (15). However, these studies are all based on outdated assessment tools not designed to measure PGD according to the DSM-5-TR and ICD-11 criteria. Therefore, researchers recommend the use of validated instruments that capture both criteria sets to assess the prevalence and determine which PGD_ICD-11_ algorithm leads to the greatest concordance with PGD_DSM-5-TR_ (15).

As demonstrated, the information on prevalence rates strongly depends on the chosen diagnostic algorithm. However, accurate estimates of prevalence rates are necessary to understand the health burden and then allocate economic and professional resources accordingly. When estimating the prevalence of PGD, not only the number of symptoms present might be decisive, but also the application of the time or duration criterion. The DSM-5-TR specifies that the loss of a loved one must have occurred at least 12 months ago before a diagnosis can be made. In ICD-11, however, the grief reaction must persist for an atypically long period of time following the loss, with a minimum of 6 months. This wording indicates an alternative application of the duration criterion, that is, that the grief response in itself and not just the bereavement period should last for at least six months. However, in some studies, only the time since loss was assessed, while in others, it is unclear whether the duration of symptoms or simply the time since the loss was assessed (e.g., 16–18).

To avoid medicalizing normal grief and overestimating the prevalence of PGD (19), determining the optimal diagnostic threshold for PGD_ICD11_ is essential (20). The aims of the current study were therefore: 1) to estimate the probable point prevalence of PGD according to the DSM-5-TR and ICD-11 criteria using a validated instrument designed to capture both criteria sets and examine the diagnostic agreement, 2) to assess the frequency of occurrence of each symptom of prolonged grief, 3) to investigate the difference between time since loss and symptom duration for a PGD_ICD-11_ diagnosis and 4) to determine which PGD_ICD-11_ algorithm results in the greatest concordance with PGD_DSM-5-TR_ when varying the number of required accessory symptoms for a PGD_ICD-11_ diagnosis in a representative sample of the population in Germany.

## Materials and methods

### Participants and procedures

Data from a representative sample of the population in Germany was collected between October and December 2021 with the assistance of a demographic consulting company (USUMA, Berlin, Germany). The sample size was determined by the ADM sampling consortium (ADM Arbeitskreis Deutscher Markt- und Sozialforschungsinstitute e.V.) that provides a sampling frame that allows to create representative samples of private households and the people living in them. The random sampling procedure involved three stages: 1) random selection of 258 regional sample point areas representing different regions of the country, 2) random selection of target households within those sample point areas using the random-route procedure, 3) random selection of one target member within target households based on a Kish-selection-grid. Inclusion criteria for target persons were age equal or above 16 years, sufficient fluency in the German language, and written informed consent. The multi-stage sampling design ensured that households were selected with equal probability. Design weighting equalized the selection probabilities within households. Distortions due to non-response were corrected by weighting adjustments. At household level, the distribution was adjusted to the population and at the individual level, further weighting was carried out to correct for biases based on the characteristics age, gender and geographic region. Regarding these characteristics the sample was representative compared to the German microcensus. The microcensus is a representative survey based on 1% of the German population (approximately 810,000 Germans), which is used for political decision-making in Germany (21).

A total of 5,901 target persons were approached by one of 198 trained interviewers. If the target person was not at home, a maximum of three further attempts were made to contact the selected person. Reasons for non-response were: a) household could not be reached (n = 791, 13.4%), b) household declined participation (n = 1,374, 23.3%), c) target person could not be reached (n = 288, 4.9%), d) target person was absent (n = 61, 1.0%), e) target person was ill or unable to follow the interview (n = 75, 1.3%), f) target person declined participation (n = 786, 13.3%). Seventeen interviews (0.3%) were not applicable for analyses. Interviews were scheduled with the remaining 2,509 participants (42.5%). The participants received oral and written information about the study and provided written informed consent. Additional parental informed consent was acquired for target persons under the age of 18. Face-to-face interviews were conducted to assess sociodemographic information. Thereafter, participants completed self-report questionnaires, and interviewers provided assistance in case of questions. The study and the procedures were approved by the local ethical review board (Leipzig University, Medical Faculty; AZ: 298/21-ek, 12.7.2021) and conducted in following the declaration of Helsinki.

### Measures

Sociodemographic data included age, gender, education, monthly household income and employment status. Bereaved participants were further asked to provide information on the characteristics of the deceased and the loss (e.g., relationship to the deceased, time since loss, cause of death). The cause of death was categorized as violent if participants indicated losing their loved one by suicide, homicide or accident. In the case of multiple losses, the participants were asked to refer to the person whose death had affected them the most.

Grief symptoms were assessed using the German version of the Traumatic Grief Inventory – Self Report Plus (TGI-SR+), which contains 22 items about grief reactions (22). All items can be found in **Table 2**. The TGI-SR+ assesses the criteria sets for PGD as defined in the DSM-5-TR and the ICD-11 (as well as the former criteria, for example PCBD). Items are rated on a 5-point Likert Scale from 1 = never to 5 = always. The TGI-SR+ has been demonstrated to be reliable and valid (22). Cronbach’s alpha in the present study indicated excellent internal consistency for the TGI-SR+ (α = .97).

**Table 2:** Frequency of occurrence of single symptoms of PGD.

	Item	DSM-5-TR	ICD-11	% (n)
1.	I had intrusive thoughts or images related to the person who died.	B2	B2	30.7 (326)
2.	I experienced intense emotional pain, sadness, or pangs of grief.	C4*	C1	20.7 (219)
3.	I found myself longing or yearning for the person who died.	B1	B1	23.4 (249)
4.	I experienced confusion about my role in life or a diminished sense of self.	–	–	9.9 (105)
5.	I had trouble accepting the loss.	–	C6	23.3 (247)
6.	I avoided places, objects, or thoughts that reminded me that the person I lost has died.	C3	–	12.4 (132)
7.	It was hard for me to trust others.	–	–	5.4 (58)
8.	I felt bitterness or anger related to his/her death.	C4*	C3	11.2 (119)
9.	I felt that that moving on (e.g., making new friends, pursuing new interests) was difficult for me.	C5	C10	7.4 (78)
10.	I felt emotionally numb.	C6	C9	14.1 (149)
11.	I felt that life is unfulfilling or meaningless without him/her.	C7	–	12.6 (134)
12.	I felt stunned, shocked, or dazed by his/her death.	–	–	19.6 (208)
13.	I noticed significant reduction in social, occupational, or other important areas of functioning (e.g., domestic responsibilities) as a result of his/her death.	D	E	8.9 (94)
14.	I had intrusive thoughts and images associated with the circumstances of his/her death.	–	–	15.6 (165)
15.	I experienced difficulty with positive reminiscing about the lost person.	–	–	8.2 (87)
16.	I had negative thoughts about myself in relation to the loss (e.g., thoughts about self-blame).	–	C2	4.8 (51)
17.	I had a desire to die in order to be with the deceased.	–	–	3.7 (39)
18.	I felt alone or detached from other individuals.	C8	–	8.1 (86)
19.	It felt unreal that he/she is dead.	C2	C4	17.7 (188)
20.	I put an intense blame on others because of his/her death.	–	C5	5.1 (54)
21.	It felt as if a part of me has died along with the deceased.	C1	C7	12.3 (131)
22.	I had difficulties experiencing positive feelings.	–	C8	9.7 (103)

Percentages were calculated from valid cases.*These symptoms are assessed with two items, the highest score of one of the two is used to tap the symptom.

To meet DSM-5-TR criteria for PGD, at least one of the two Criterion B symptoms, at least three of the eight Criterion C symptoms, and the Criterion D symptom (i.e., functional impairment) must be endorsed for those who experienced the death of a loved one at least 12 months prior (Criterion A) (9, 10). All Criterion C symptoms are tapped by one item, except for one symptom (C4: “Intense emotional pain (e.g., anger, bitterness, sorrow) related to the death”), which is captured by two items. The highest score on one of these two items was used to represent the C4 criterion. The time criterion of 12 months was assessed with the following question: “How much time has passed since the loss? (In Months)”. All participants who indicated at least 12 months were counted.

To meet ICD-11 criteria for PGD, at least one of the two Criterion B symptoms, at least one Criterion C symptom, the Criterion D symptom (i.e., functional impairment) and the Criterion E (the grief response has persisted for at least six months) should be endorsed for those who experienced the death of a loved one (Criterion A).

The duration criterion E was assessed with the following question: “Have you had the feelings described above for at least six months?”.

### Statistical analyses

All statistical analyses were conducted using the Statistical Package for Social Sciences, version 27 (IBM® SPSS®). The significance level was set to α = .05.

To estimate the prevalence of PGD, the number of participants fulfilling the criteria described above was counted. A symptom was considered present if scores were ≥ 4 (at least ‘often’) (22). Each symptom was dichotomously coded as ‘not present’ (0) or ‘present’ (1). For exploratory reasons, percentages of endorsement of each item were calculated.

To examine the difference between PGD_DSM-5-TR_ and PGD_ICD-11,_ Fisher’s exact test was used. Pairwise agreement between both diagnostic algorithms was evaluated using kappa statistics with a 95% confidence interval. To investigate the difference between time since loss and symptom duration for a PGD_ICD-11_ diagnosis, the duration criterion E was applied in two different ways (time since loss = 6 months vs. symptom duration = 6 months). Fisher’s exact test was again used to test statistical significance. Furthermore, the diagnostic rates of PGD_ICD-11_ were calculated with an increasing number of required accessory symptoms from 2+ to 7+ symptoms, and pairwise agreement with PGD_DSM-5-TR_ was evaluated again using kappa statistics.

## Results

Of the 2,509 participants between the ages of 16 and 95 years, 50.9% were female. The mean age was 49.48 years (SD=17.81). **Table 3** provides an overview of the participant characteristics. Of all participants, 1,071 (54.2%) reported having experienced the loss of a close person (e.g., a partner, relative or good friend). Nine participants had to be excluded due to missing data leading to a final data set of n=1,062. Bereaved individuals were primarily middle-aged, and 55.6% were female. The majority reported having lost a parent (40.9%), or other relatives besides children, parent, or partner (28.8%), and natural nonviolent deaths were reported most frequently (89.0%) as the cause of death (see **Table 4**).

**Table 3:** Demographic characteristics of the sample.

	Total sample (N=2509)	Bereaved sample (n=1062)
Gender		
Female, n (%)	1276 (50.9)	590 (55.6)
Male, n (%)	1230 (49.0)	471 (44.3)
Divers, n (%)	3 (0.1%)	1 (0.1%)
Age, M (SD)	49.48 (17.81)	56.52 (16.93)
Education		
Primary, n (%)	669 (26.7)	357 (33.6)
Secondary, n (%)	1229 (49.0)	464 (43.7)
Tertiary, n (%)	565 (22.5)	230 (21.7)
In school, n (%)	46 (1.8)	11 (1.0)
Employment status		
Employed, n (%)	1537 (61.3)	549 (51.8)
Unemployed, n (%)	189 (7.5)	83 (7.8)
Retired, n (%)	622 (24.8)	398 (37.5)
In training/education	156 (6.2)	30 (2.8)
Monthly household net income		
< 1250 EUR, n (%)	283 (11.4)	128 (12.2)
1250-2500 EUR, n (%)	956 (38.6)	470 (44.7)
≥ 2500 EUR, n (%)	1237 (50.0)	453 (43.1)

Percentages were calculated from valid cases.

**Table 4:** Characteristics of the bereaved sample (n=1062).

Time since the death in months, M (SD)	102.88 (124.57)
< 6 months, n (%)	63 (5.9)
6-12 months, n (%)	79 (7.5)
1-2 years, n (%)	117 (11.0)
2-5 years, n (%)	276 (26.1)
5-10 years, n (%)	208 (19.6)
> 10 years, n (%)	316 (29.8
Deceased is	
Partner, n (%)	184 (17.4)
Child, n (%)	28 (2.6)
Parent, n (%)	433 (40.9)
Other relative, n (%)	305 (28.8)
Friend, n (%)	109 (10.3)
Cause of death	
Natural, nonviolent, n (%)	944 (89.0)
Unnatural, violent, n (%)	117 (11.0)

Percentages were calculated from valid cases.

The conditional prevalence of PGD using the DSM-5-TR diagnostic algorithm was 4.7% (n = 50), within the population-based sample (including non-bereaved), 2.0%. Using the diagnostic algorithm of the ICD-11 led to a conditional prevalence of PGD of 5.4% (n=57), within the population-based sample (including non-bereaved) 2.3%.

**Figure 1:**
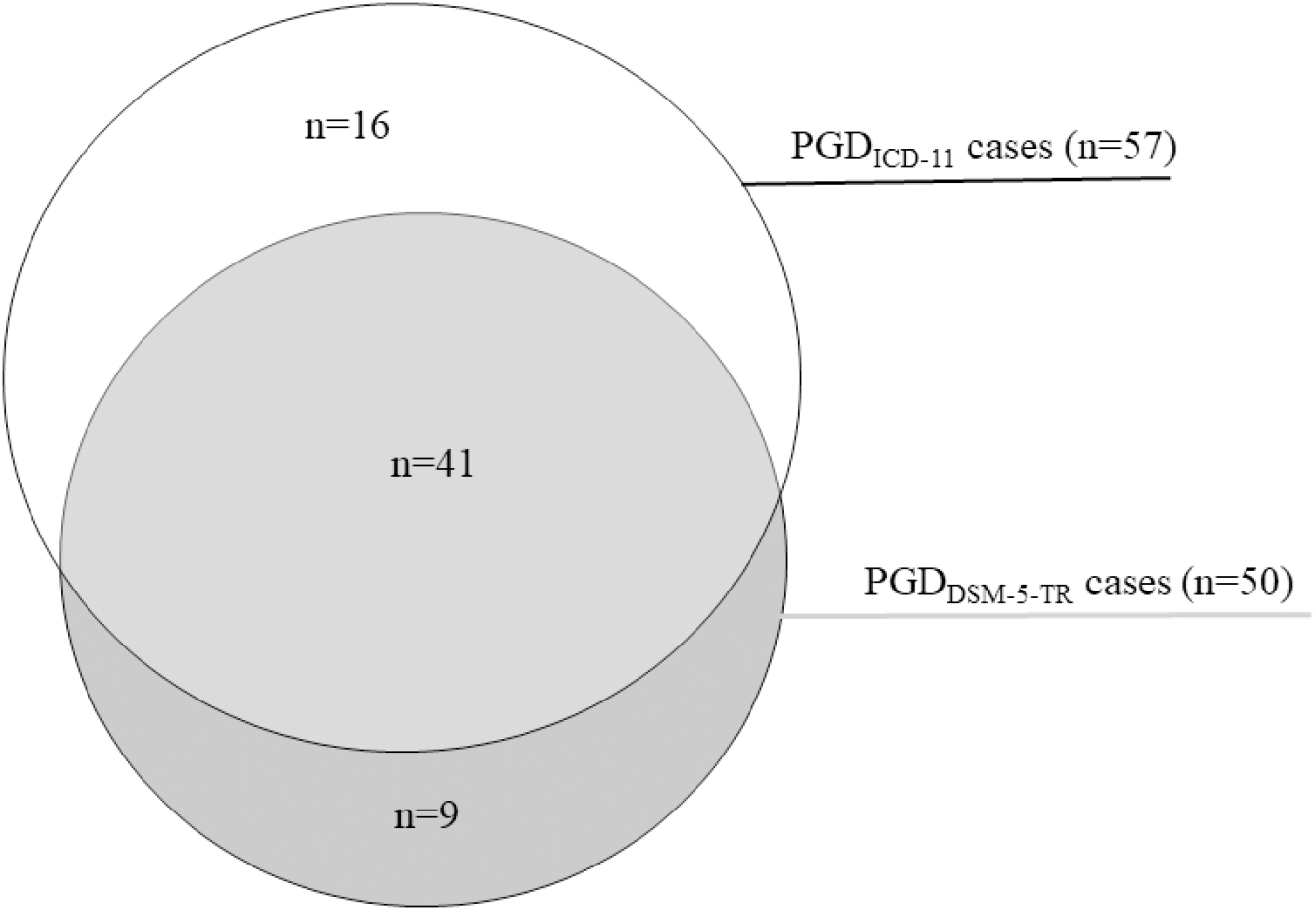
PGD_DSM-5-TR_ cases vs. PGD_ICD-11_ cases.

The PGD_DSM-5-TR_ diagnostic rate was significantly lower than the rate of PGD_ICD-11_ (Fisher’s exact test, *p* <.001). The pairwise agreement between PGD_DSM-5-TR_ and PGD_ICD-11_ was substantial with κ = 0.75, 95% CI [0.66–0.85]. There were nine unique PGD_DSM-5-TR_ cases (i.e., meeting PGD_DSM-5-TR_ but not PGD_ICD-11_ criteria) and 16 unique PGD_ICD-11_ cases (i.e., meeting PGD_ICD-11_ but not PGD_DSM-5-TR_ criteria). The remaining 41 participants (3.9%) met both PGD criteria (see **Figure 1**).

The most frequently indicated symptoms of the TGI-SR+ were having intrusive thoughts or images related to the person who died (30.7%), followed by longing or yearning for the person who died (23.4%), having trouble accepting the loss (23.3%) and feeling stunned, shocked, or dazed by his/her death (19.6%; see **Table 2**). Functional impairment due to the death was reported by 8.9% (n=94).

**Figure 2:**
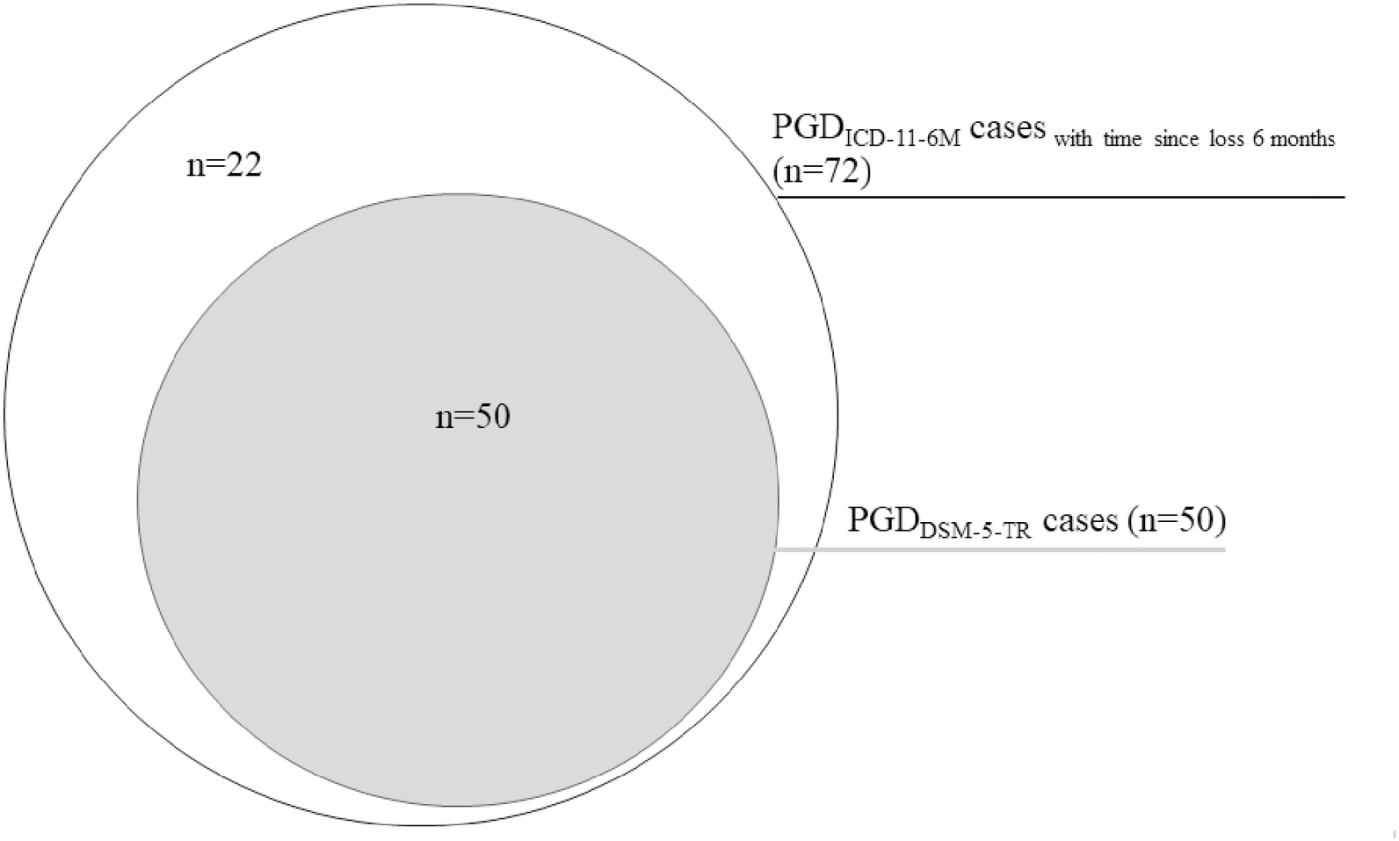
PGD_DSM-5-TR_ cases vs. PGD_ICD-11-6M_ cases.

When simply examining time since the loss (i.e., six months) instead of symptom duration, the prevalence of PGD using the ICD-11 criteria B, C, and D, here referred to as PGD_ICD-11-6M_ increased to 6.8% (n=72), which is significantly higher than the rate of 5.4% for PGD_ICD-11_ (Fisher’s exact test, *p* <.001) and higher than the rate of 4.7% for PGD_DSM-5-TR_ (Fisher’s exact test, *p* <.001). Within the population-based sample (including non-bereaved), the prevalence increased to 2.9%. The pairwise agreement between PGD_DSM-5-TR_ and PGD_ICD-11-6M_ with time since loss six months was substantial with κ = 0.81, 95% CI [0.73-0.89]. There were no unique PGD_DSM-5-TR_ cases and 22 unique PGD_ICD-11-6M_ cases. The remaining 50 participants (4.7%) met both PGD criteria (see **Figure 2**).

**Figure 3:**
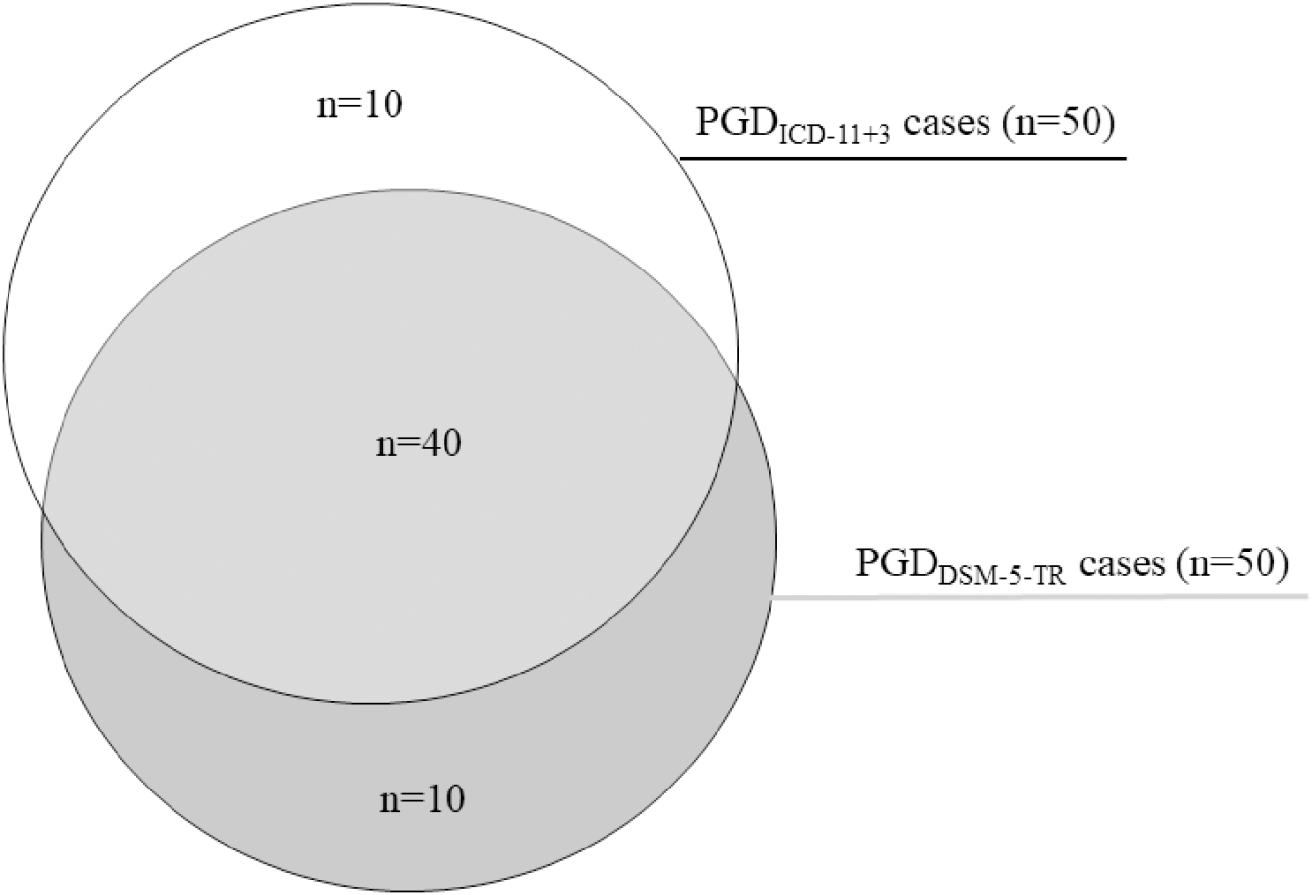
PGD_DSM-5-TR_ cases vs. PGD_ICD-11+3_ cases with 3 additional symptoms.

The number of required diagnostic criteria for PGD_ICD-11_ was subsequently increased in additional analyses. The conditional prevalence decreased from 5.4% with one accessory symptom to 0.9% with seven accessory symptoms (see **Table 5**). The highest pairwise agreement with PGD_DSM-5-TR_ was achieved when the number of accompanying symptoms was increased to 3 with κ = 0.79, 95% CI [0.70-0.88]. Increasing the number of accompanying symptoms to 3 for a PGD_ICD-11_ diagnosis led to a prevalence of 4.7% (n=50). There were 10 unique PGD_DSM-5-TR_ cases and 10 unique PGD_ICD-11_ cases in this case. The remaining 40 participants (3.8%) met both PGD criteria (see **Figure 3**).

**Table 5:** The prevalence rate of PGD_ICD-11_ with increasing numbers of accessory symptoms and pairwise agreement with PGD_DSM-5-TR_.

Number of accessory symptoms	Probable ICD-11 cases		Pairwise agreement with PGD_DSM-5-TR_ cases (4.7%, n=50)
	%	(n)	kappa
PGD 1+	5.4	57	κ= 0.75
			CI: 0.66-0.85
PGD 2+	5.3	56	κ=0.76
			CI:0.67-0.85
PGD 3+	4.7	50	κ=0.79
			CI: 0.70-0.88
PGD 4+	3.9	41	κ=0.76
			CI: 0.66-0.86
PGD 5+	2.6	28	κ=0.66
			CI: 0.53-0.78
PGD 6+	1.9	20	κ=0.50
			CI: 0.36-0.64
PGD 7+	0.9	10	κ=0.29
			CI: 0.14-0.44

## Discussion

One aim of this study was to estimate the probable point prevalence of PGD using a validated instrument designed to capture the DSM-5-TR and ICD-11 criteria sets and evaluate their diagnostic agreement in a population-based sample. Previous prevalence estimates were predominantly based on old criteria sets or outdated assessment tools. Previous studies also showed little content overlap between diagnostic criteria and found varying prevalence rates. Determining the optimal diagnostic threshold seems essential to avoid medicalizing normal grief and overestimating the prevalence of PGD.

The first main finding was a conditional prevalence of PGD_DSM-5-TR_ of 4.7%, which was significantly lower than the rate of PGD_ICD-11_ of 5.4%. These estimates are slightly higher than the ones found in other population-based studies by Rosner et al. (PGD_DSM-5-TR_ 3.3%, PGD_ICD-11_ 4.2%) (14) and Treml et al. (PGD_DSM-5-TR_ 3.4%) (23). These differences might emerge from the fact that both prior studies used outdated instruments designed for previous diagnostic sets, thus not assessing all criteria, while the TGI-SR+ used in the current study was specifically designed to capture all current diagnostic criteria sets. The pairwise agreement between criteria sets in the present study was substantial (κ = 0.75), with 3.9% of the bereaved meeting both PGD criteria. However, there were still nine unique PGD_DSM-5-TR_ cases and 16 unique PGD_ICD-11_ cases, demonstrating that there is a need for further convergence of the diagnostic criteria and their algorithm.

Another aim was to assess the frequency of occurrence of each symptom of prolonged grief. The advantage of the TGI-SR+ is that it captures not only the current diagnostic criteria but also former criteria for PGD such as PCBD or the criteria set proposed by Prigerson in 2009 (PGD_2009_), on which much research was based (4). The most frequently indicated symptoms were having intrusive thoughts or images related to the person who died (criterion B2, 30.7%) and longing or yearning for the person who died (criterion B1, 23.4%), which are considered the core criteria for PGD (15). The core criteria were followed by having trouble accepting the loss (23.3%). The last-mentioned symptom, however, is only a criterion within the ICD-11 and not listed within the DSM-5-TR, even though it was listed within all previous diagnostic criteria sets (for an overview see 15). Since this criterion is frequently reported, it could contribute to the higher prevalence rate of PGD_ICD-11_ compared to PGD_DSM-5-TR_. The next most common symptom, reported by nearly 20%, was feeling stunned, shocked, or dazed by his/her death. This symptom originates from the previous PGD_2009_ diagnostic criteria by Prigerson (4). In their study, 19.2% indicated feeling stunned, shocked, or dazed about the death, which led the authors to propose this symptom as a diagnostic criterion for PGD. Every study using for instance the PG-13 or the Inventory of Complicated Grief (ICG) subsequently assessed this item as one of the diagnostic criteria. However, the symptom was later not included in the ICD-11 or DSM-5-TR. Some studies used the item “feeling stunned, shocked, or dazed by his/her death” to assess emotional numbness (e.g., 14). In the current study, only 14% reported emotional numbness, indicating that these symptoms might be similar but do not assess the same feelings.

The third aim was to investigate the difference between the time since loss and symptom duration for a PGD_ICD-11_ diagnosis. The prevalence rate of PGD_ICD-11_ was significantly higher when only the bereavement period rather than the symptom duration was examined (6.8% vs 5.4%). In this case, the diagnostic agreement between PGD_ICD-11-6M_ and PGD_DSM-5-TR_ increased (κ = 0.81), since all individuals meeting the DSM-5-TR criteria were also enclosed when assessing the PGD_ICD-11-6M_ criteria, and there were no unique PGD_DSM-5-TR_ cases (see **Figure 2**). However, this algorithm led to 22 unique PGD_ICD-11-6M_ cases and a high prevalence rate of 6.8%. This prevalence rate is likely an overestimation of the true prevalence since all people bereaved for six months who meet the minimum criteria according to ICD-11 are included, even those whose symptoms have persisted for a shorter period of time. This finding indicates that the bereavement period alone might not be a reliable indicator for PGD but rather the symptom duration. Only considering bereaved with symptoms for at least six months leads to a much smaller and presumably more precise estimate that corresponds more closely to the DMS-5-TR. Nevertheless, the intersection between PGD_ICD-11_ and PGD_DSM-5-TR_ still needs improvement. For the clinical practice, we recommend assessing the symptom duration as a vital part in diagnosing PGD instead of simply assessing the bereavement period.

The last aim was to determine which PGD_ICD-11_ algorithm results in the greatest concordance with PGD_DSM-5-TR_ when varying the number of required accessory symptoms for a PGD_ICD-11_ diagnosis. The results show that the highest agreement with PGD_DSM-5-TR_ was achieved when the number of accompanying symptoms for a PGD_ICD-11_ diagnosis was increased to 3, which resulted in a lower prevalence of 4.7%. This result aligns with other studies that demonstrated that the minimal criteria of PGD_ICD-11_ might be too liberal, as they lead to relatively high prevalence rates (20, 24), and a more conservative scoring rule might be beneficial. For instance, Rosner and colleagues found the highest agreement between both criteria sets with four accessory symptoms (14), while others recommend even five or more accessory symptoms for a PGD_ICD-11_ diagnosis (22, 24). Increasing the accompanying symptoms for a PGD_ICD-11_ diagnosis leads to a better agreement with PGD_DSM-5-TR,_ and in our case, even to the same prevalence rate. Nonetheless, there were still 10 unique PGD_DSM-5-TR_ cases and 10 unique PGD_ICD-11_ cases, indicating that there is still variability within the groups of people meeting grief disorder criteria. A less heterogeneous diagnosis would be more beneficial for clinical practice and research.

Taken together, our results have implications for clinical practice. PGD is a serious disorder in the general population that is associated with significant impairments. Healthcare providers should be aware of the diagnosis and screen for it if indicated. However, this should be done using up-to-date measurement tools that capture the current diagnostic criteria, such as the TGI-SR+. When diagnosing PGD, the symptom duration, in particular, should be taken into account. Furthermore, the minimum criteria according to ICD-11 seem to be too liberal, as they lead to relatively high prevalence rates, and a more conservative scoring rule seems appropriate in order to avoid misdiagnosis.

Our findings have to be interpreted in light of some limitations. First, our results are based on self-reported data rather than clinician-administered structured interviews. The exclusive use of self-report measures could have led to bias due to misinterpretation of questions. Furthermore, a cross-sectional design was applied, eliminating any causal conclusions and conclusions about the stability of prolonged grief symptoms. Since grief naturally fluctuates in response to external stressors (e.g., anniversaries, birthdays, holidays), a longitudinal approach using multiple assessments beyond six and twelve months might be more appropriate to avoid diagnosing temporary distress. In our study, the data was collected between October and December, i.e. in some cases shortly before Christmas, which could be such an external stressor. However, the question of whether the symptoms have persisted for at least six months may provide some indication of symptom stability. Additionally, our results regarding the prevalence might be specific to the German general population. Hence, attempts should be made to replicate the findings in different cultures, in order to confirm the generalizability of our findings to other populations. Lastly, general mental health problems and psychiatric comorbidities were not examined. Therefore, the extent to which possible comorbid psychopathology influenced the results is unknown, challenging the interpretation of the results.

A major strength of the current study is the population-based setting, with a sample constructed to be representative in terms of age, gender, and education. The representativeness of the sample is especially relevant since most studies on PGD include predominately female participants leading to an underrepresentation of men.

The results demonstrate that the prevalence of PGD varies significantly depending on the application of the diagnostic algorithm and criteria. The prevalence ranged from 4.7% for PGD_DSM-5-TR_ to 5.4% for PGD_ICD-11_ to even 6.8% for PGD_ICD-11-6M_ when only considering the bereavement period. Yet, at the same time, the diagnostic agreement between all sets was substantial. Increasing the accessory symptoms for a PGD_ICD-11_ diagnosis to 3 lowered the prevalence to 4.7%, which is equivalent to the DSM-5-TR prevalence. However, the overlap between the two systems could to be improved by harmonizing the criteria. PGD remains a substantial disorder among the bereaved and should not be neglected by healthcare providers.

## Data availability statement

The raw data supporting the conclusions of this article will be made available by the authors, without undue reservation.

## Ethics statement

The study and the procedures were approved by the local ethical review board (Leipzig University, Medical Faculty; AZ: 298/21-ek, 12.7.2021). The studies were conducted in accordance with the local legislation and institutional requirements. Written informed consent for participation in this study was provided by the participants themselves or by legal guardians/next of kin in case of minors.

## Author contributions

JT: Conceptualization, Data curation, Formal analysis, Investigation, Methodology, Validation, Visualization, Writing – original draft, Writing – review & editing. KL: Data curation, Formal analysis, Methodology, Writing – review & editing. EB: Investigation, Methodology, Validation, Writing – review & editing. AK: Conceptualization, Resources, Supervision, Validation, Writing – review & editing.
